# Effects of Dietary Vitamin D Levels on Markers Related to Amyloidogenesis and Neuroinflammation in db/db Mice

**DOI:** 10.3390/nu17213339

**Published:** 2025-10-24

**Authors:** Jisu Kim, Dain Wi, Sung Nim Han, Chan Yoon Park

**Affiliations:** 1 Department of Food and Nutrition, The University of Suwon, Hwaseong 18323, Republic of Korea; 2 Department of Food and Nutrition, College of Human Ecology, Seoul National University, Seoul 08826, Republic of Korea; 3 Research Institute of Human Ecology, College of Human Ecology, Seoul National University, Seoul 08826, Republic of Korea

**Keywords:** vitamin D, Alzheimer’s disease, type 2 diabetes, amyloidogenesis, neuroinflammation

## Abstract

Background/Objectives: Low vitamin D levels are associated with an elevated risk of Alzheimer’s disease (AD). Given the rising prevalence of diabetes and its association with AD, this study investigated whether vitamin D modulates amyloidogenesis and inflammation in the brains of diabetic mice. Methods: Five-week-old male C57BLKS/J-*m+/m+*(con) and C57BLKS/J-*db/db* (db) mice received diets with low or high vitamin D (LVD or HVD) for 8 weeks. Hippocampal neuronal morphology was assessed using H&E and Nissl staining, and Aβ levels, along with the mRNA expression of genes related to amyloidogenesis, amyloid degradation, inflammation, antioxidation, and neurotrophic factors, were measured in the hippocampus and prefrontal cortex (PFC). Results: High dietary vitamin D levels attenuated neuronal necrosis in db/db mice. Hippocampal *App* and *Bace1* expression levels were higher in db/db mice; however, amyloidogenic gene (*App*, *Bace1*, *Ps1*) expression levels in both the hippocampus and PFC were significantly lower in db_HVD group compared with those in db_LVD group (all *p* < 0.05). Among control mice, PFC *App* and *Ps1* expression levels were lower in con_HVD group than in con_LVD group. Nonetheless, Aβ42 protein levels were not affected by either diabetes or dietary vitamin D levels. Furthermore, lower hippocampal *Iκbα* and PFC *Mcp-1* expression levels in db_HVD group than those in db_LVD group were observed, both upregulated in diabetic mice. Amyloid degradation-related gene or *Vdr* expression was not altered by dietary vitamin D levels. Conclusions: These findings suggest that vitamin D may exert neuroprotective effects on the hippocampus and PFC in diabetic mice by mitigating neuronal damage and suppressing amyloidogenic and inflammatory gene expression.

## 1. Introduction

Alzheimer’s disease (AD) is the most prevalent neurodegenerative disorder, characterized by progressive cognitive decline. According to the 2022 World Alzheimer Report, more than 55 million people worldwide were living with dementia, including AD, in 2019, and this number is expected to rise to 139 million by 2050 [1]. This trend underscores the urgent need for developing both preventive and therapeutic strategies for AD. A key pathological feature of AD is the accumulation of amyloid-beta (Aβ) and the presence of neuroinflammation [2]. Aβ accumulation involves the release of Aβ peptides into the extracellular space, where they aggregate into insoluble amyloid plaques. These plaques form around neurons, exert neurotoxic effects, activate inflammatory cascades, and ultimately lead to neuronal death [3]. Beyond Aβ aggregation, neuroinflammation and regional brain atrophy in the hippocampus are reported as early pathological features of AD [4,5]. Furthermore, chronic diseases such as type 2 diabetes mellitus (T2DM), which become more prevalent with aging, have been associated with the early pathology of AD, emphasizing its multifactorial etiology.

T2DM, a metabolic disorder characterized by insulin resistance and hyperglycemia, has emerged as a significant risk factor for cognitive decline and AD [6]. A 12-year prospective population-based study demonstrated that older adults with T2DM face at least a two-fold increased risk of developing AD compared with those without [7]. Additionally, a meta-analysis found diabetes to be associated with a 1.25–1.91-fold increase in the risk of cognitive impairment and dementia [8]. These epidemiological findings imply that altered insulin signaling or neuroinflammation induced by T2DM may contribute to AD pathogenesis. Insulin can enter the brain through receptor-mediated transport or transcytosis across the blood–brain barrier (BBB). This process has been reported to be attenuated under metabolic and inflammatory conditions, such as obesity and diabetes, through receptor downregulation and endothelial dysfunction [9,10,11]. In T2DM, systemic insulin resistance has been shown to impair central nervous system (CNS) insulin signaling, activating multiple downstream pathways, including the mitogen-activated protein kinase (MAPK) and PI3K/Akt cascade [12,13,14]. These alterations enhance neuroinflammation by increasing the production of proinflammatory cytokines and chemokines. Moreover, T2DM compromises BBB integrity, facilitating the entry of toxic substances and inflammatory mediators into the brain. Such changes may exacerbate neuronal damage, ultimately accelerating AD progression [15].

Vitamin D, a fat-soluble secosteroid hormone, is primarily recognized for its essential role in bone health and mineral homeostasis [16]. Beyond these classical functions, vitamin D exhibits immunomodulatory properties, including anti-inflammatory and antimicrobial effects [17,18], which are particularly relevant in the CNS [19,20]. These effects are supported by the presence of vitamin D receptors (VDRs) and hydroxylases that activate vitamin D in various brain regions, including the hippocampus, prefrontal cortex (PFC), and hypothalamus [21]. Furthermore, since vitamin D can traverse the BBB, it has been reported to exert neuroprotective effects in the brain by alleviating neuroinflammation and oxidative stress, as well we by enhancing the production of neurotrophic factors [22]. Specifically, patients with serum 25(OH)D levels below 25 ng/mL had an increased risk of developing AD compared with those with levels above 25 ng/mL [23]. Furthermore, a 7-year follow-up study indicated that higher dietary vitamin D intake was associated with a lower risk of developing AD among older women [24].

In addition to its functions in the brain, vitamin D regulates glucose metabolism and systemic inflammation, suggesting its potential role in metabolic disorders [25,26]. Vitamin D status was inversely associated with diabetes incidence in NHANES data for non-Hispanic White and Mexican American populations [27], and positively correlated with insulin sensitivity and pancreatic β-cell function in a healthy cohort [28]. Impaired innate immunity observed in diabetes can lead to hyperinflammation, which may further exacerbate diabetic complications, whereas vitamin D has been reported to enhance natural killer cell activity and mitigate inflammation in diabetes [29]. Furthermore, maintaining sufficient vitamin D levels has been associated with an approximately 55% lower risk of T2DM [30].

Diabetic conditions exacerbate early AD hallmarks, including Aβ accumulation, tau hyperphosphorylation, and neuroinflammation [31]. Vitamin D reportedly counteracts these processes by regulating inflammatory cytokines [32], improving glucose metabolism [33], and reducing oxidative stress [34] both in the brain and peripheral tissues. These findings suggest that dietary vitamin D levels may mitigate AD-related pathology, particularly under diabetic conditions. Based on this evidence, the present study investigated whether high dietary vitamin D levels could attenuate early AD pathology in db/db mice, focusing on amyloidogenesis, Aβ degradation, inflammatory markers, and neurotrophic factors in the hippocampus and PFC.

## 2. Materials and Methods

### 2.1. Animals and Diets

Five-week-old male C57BLKS/J-*m+/m+* and C57BLKS/J-*db/db* mice were purchased from SLC Japan, Inc. (Shizuoka, Japan). All mice were housed in a semi-specific pathogen-free animal facility at Seoul National University. After a 3-day acclimation period, the mice were randomly assigned to four diet-based experimental groups and fed for 8 weeks: (1) con_LVD: C57BLKS/J-*m+/m+* mice fed a 10% fat normal diet with 948 IU vitamin D/kg diet (n = 13), (2) con_HVD: C57BLKS/J-*m+/m+* mice fed a 10% normal diet with 9477 IU vitamin D/kg diet (n = 14), (3) db_LVD: C57BLKS/J-*db/db* mice fed a 10% fat normal diet with 948 IU vitamin D/kg diet (n = 10), and (4) db_HVD: C57BLKS/J-*db/db* mice fed a 10% normal diet with 9477 IU vitamin D/kg diet (n = 10). The composition of the experimental diets is presented in Table S1. All animal procedures were approved by the Institutional Animal Care and Use Committee of Seoul National University (approval number: SNU-220907-2-1, approval date: 7 September 2022).

### 2.2. Blood and Tissue Collection

At the end of the 8-week experimental period, animals were subjected to a 12 h fasting period then euthanized via CO_2_ asphyxiation. Blood samples and brain tissues were collected. Thereafter, the brains were dissected into two hemispheres and further subdivided into the hippocampus, PFC, and thalamus, all of which were also stored at −80 °C for subsequent experiments (Figure S1). For histological analysis, whole brains from selected mice in each group were dissected and immediately fixed in 20% neutral buffered formalin.

### 2.3. Hematoxylin and Eosin (H&E) Staining and Nissl Staining

After fixation, the brain samples (n = 2–3 animals/group) were embedded in paraffin wax for the histological analysis of hippocampal neurons. The paraffin-embedded tissues were sectioned to a thickness of 4 μm and subjected to histological examination using H&E and Nissl staining to assess histopathological changes in the CA1 region of the hippocampus across the experimental groups. The sections were stained with Harris hematoxylin solution (BBC Biochemical, Washington, DC, USA), eosin Y solution (Sigma-Aldrich, St. Louis, MO, USA), and 0.1% Cresyl Echt Violet Solution (ScyTek Lab, Logan, UT, USA). Stained sections were subsequently examined under a microscope and photographed.

### 2.4. Total RNA Extraction, cDNA Synthesis, and qRT-PCR

Total RNA was extracted from the hippocampus and PFC of left hemisphere brain tissues of mice (n = 10 for con_LVD and con_HVD; n = 7 for db_LVD and db_HVD), using RNAiso Plus (Takara, Singa, Japan) according to the manufacturer’s instructions. The purity and concentration of the RNA were assessed using a Microvolume Spectrophotometer (DeNovix, Wilmington, DE, USA). The extracted RNA was subsequently reverse-transcribed into cDNA using the PrimeScript™ II First Strand cDNA Synthesis Kit (Takara, Singa, Japan) and amplified using the Light Cycler 96 (Roche, Mannheim, Germany) with TB Green Premix Ex Taq (Takara, Shiga, Japan) to evaluate the relative expression levels of target genes. Glyceraldehyde-3-phosphate dehydrogenase (*Gapdh*) served as the internal standard to normalize the expression levels of all target genes. The analyzed genes included those involved in Aβ production (*App*, *Bace1*, and *Ps1*) and degradation (*Adam10*, *Ide*, and *Nep*), inflammatory responses (*IκBα*, *Tnf-α*, *Il-6*, *Mcp-1*, *Ccl5,* and *Cx3cl1*), antioxidant responses (*Nrf2* and *Ho-1*), vitamin D signaling (*Vdr* and *Pdia3*), calcium homeostasis (*CaMKIIα* and *Serca2b*), and neurotrophic factors (*Ngf*, *Bdnf*, and *Nt-3*). The primer sequences for these genes are listed in Table S2.

### 2.5. Amyloid-Beta 42 (Aβ42) Protein Quantification via Enzyme-Linked Immunosorbent Assay (ELISA)

The right hippocampal regions (n = 5–6 animals/group) were homogenized in 16 volumes of 5 M guanidine–HCl/50 mM Tris-HCl (pH 8.0) and incubated for 3–4 h with gentle mixing. The homogenates were subsequently supplemented with 9 volumes of a protease inhibitor cocktail containing 4-(2-aminoethyl)-benzenesulfonyl fluoride hydrochloride (AEBSF, P-2714; Sigma-Aldrich, St. Louis, MO, USA) and centrifuged at 16,000× *g* for 20 min at 4 °C. The supernatants were subjected to ELISA for total Aβ42 according to the manufacturer’s instructions (KMB3441; Thermo Fisher Scientific, Waltham, MA, USA). Aβ42 concentrations were normalized to total protein content, measured using the bicinchoninic acid assay, and expressed as pg Aβ42 per μg total protein.

### 2.6. Western Blot

Total proteins were extracted from the right PFC using RIPA buffer (Biomax, Seoul, Korea) supplemented with a protease inhibitor cocktail containing AEBSF (P-2714, Sigma-Aldrich, St. Louis, MO, USA). The tissue extracts were separated using 15% sodium dodecyl sulfate–polyacrylamide gel electrophoresis and transferred to a polyvinylidene difluoride membrane. The membrane was blocked with 5% skim milk and incubated overnight at 4 °C with primary antibodies against Aβ42 (1:1000, #44-344; Thermo Fisher Scientific, Waltham, MA, USA) and β-actin (1:2000, D6A8; Cell Signaling Technology, Danvers, MA, USA). After washing, the membrane was incubated with horseradish peroxidase-conjugated anti-rabbit IgG secondary antibodies (Cell Signaling Technology, Danvers, MA, USA). Protein bands were visualized using West Glow FEMTO chemiluminescent substrate (Biomax, Seoul, Korea) and quantified using ImageJ software Version 1.54 (National Institutes of Health, MD, USA).

### 2.7. Statistical Analysis

All data are presented as the mean ± standard error of the mean (SEM). A two-way analysis of variance (ANOVA) was conducted to examine the effects of diabetes, amounts of dietary vitamin D levels, and their interaction across experimental groups. Additionally, one-way ANOVA followed by Fisher’s Least Significant Difference (LSD) post hoc test was used to identify statistical differences among the four groups. Statistical significance was established at *p* < 0.05. All statistical analyses were performed using SPSS (version 26; IBM SPSS Inc., Chicago, IL, USA).

## 3. Results

Table 1 presents the body weight, food intake, vitamin D intake, and blood glucose levels of the experimental animals. After 8 weeks of intervention, the body weights of the db/db mice (db_LVD and db_HVD) were approximately 1.3-fold higher, while blood glucose levels were 3.8-fold higher than those of the control mice (con_LVD and con_HVD). The average daily food intake of the db/db mice (5.2 ± 0.13 g) was approximately twice that of the control mice (2.6 ± 0.02 g). In the HVD groups, daily vitamin D intake reached 25.4 ± 0.27 IU (con_HVD) and 53.1 ± 2.69 IU (db_HVD), representing a ten-fold greater intake compared with that in the LVD groups (con_LVD, 2.6 ± 0.01 IU; db_LVD, 5.0 ± 0.12 IU). Despite significant differences in vitamin D intake among groups, dietary vitamin D levels did not significantly affect body weight, food intake, or blood glucose levels in db/db mice.

**Figure 1:**
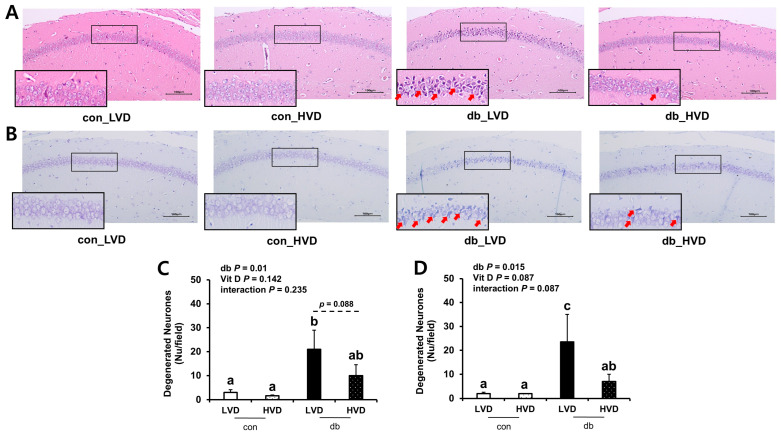
Morphology of Neuronal Necrosis in the Hippocampal CA1 Region and Quantitative Analysis of Degenerated Neurons. Representative images of (**A**) H&E staining and (**B**) Nissl staining. Red arrow indicates dead cells. Quantitative analysis of degenerated neurons is denoted by (**C**) H&E staining and (**D**) Nissl staining. Data are presented as mean values ± SEMs (n = 2–3 animals/group). *p*-values from two-way ANOVA are shown in the upper left corner of the graph to indicate the effects of diabetes, vitamin D levels, and their interaction. Different superscript letters (a, b, c) represent significant group differences (*p* < 0.05), and *p*-value on the dashed line indicate difference between two groups, as determined by Fisher’s LSD multiple-comparison test.

To ascertain whether db/db pathology and dietary vitamin D levels induced morphological alterations in hippocampal neurons within the CA1 region, brain sections were examined using H&E (Figure 1A,C) and Nissl (Figure 1B,D) staining. Both staining methods revealed a higher number of apoptotic neurons in the db/db mice compared with those in the control mice (*p* < 0.05). In particular, histological analysis demonstrated pronounced neuronal cell death in the CA1 region of the db_LVD group, evidenced by numerous red arrows marking apoptotic neurons (Figure 1A). In contrast, the db_HVD group exhibited a lower density of apoptotic neurons (Figure 1B). Quantitative analysis of Nissl-stained sections indicated that the number of apoptotic neurons in the db_LVD group (23.5 ± 11.5) was 3.4-fold higher than in the db_HVD group (7.0 ± 3.0; *p* < 0.05; Figure 1D). Similarly, H&E staining indicated a 2.1-fold greater tendency of apoptotic neurons in the db_LVD group (21 ± 8.0) than in the db_HVD group (10 ± 4.5) (*p* = 0.088; Figure 1C). These results indicate that hippocampal neurons in db/db mice may be susceptible to diabetes-induced damage, and that high dietary vitamin D level could potentially mitigate this neurodegeneration.

To determine whether neuronal damage in db/db mice and its attenuation by vitamin D were linked to Aβ pathology, we analyzed the mRNA expression of amyloidogenic and Aβ degradation-related genes in the hippocampus and PFC. In diabetic mice, the hippocampal expression of *App* and *Bace1*, which encode the amyloid precursor protein and β-secretase, respectively, was significantly upregulated, indicating enhanced amyloidogenic activity (two-way ANOVA, *p* < 0.05). However, the expression of *App* and *Ps1* in the PFC was not significantly affected by diabetes, while *Bace1* expression was significantly lower in db/db mice (two-way ANOVA, *p* < 0.05).

**Figure 2:**
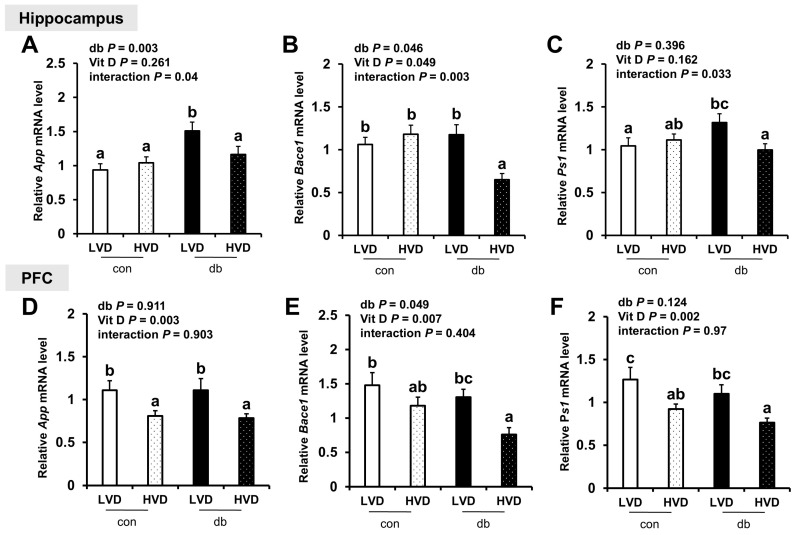
mRNA Expression Levels of Amyloidogenic Genes in the Hippocampus and PFC. The relative mRNA expression levels of *App*, *Bace1*, and *Ps1* in the hippocampus (**A**–**C**), along with those in the PFC (**D**–**F**), were measured using RT-PCR. Data are presented as mean values ± SEMs (n = 10 for con_LVD and con_HVD; n = 7 for db_LVD and db_HVD). *p*-values from two-way ANOVA are shown in the upper left corner of the graph to indicate the effects of diabetes, vitamin D levels, and their interaction. Different superscript letters (a, b, c) represent significant group differences (*p* < 0.05), as determined by Fisher’s LSD multiple-comparison test.

High dietary vitamin D level suppressed the mRNA expression of amyloidogenic genes in both regions of the brain, with significant reductions in *App*, *Bace1*, and *Ps1* (a component of γ-secretase involved in Aβ generation) in the db_HVD group compared with in the db_LVD group (Figure 2A–F, *p* < 0.05). Likewise, among control mice, *App* and *Ps1* expression levels in the PFC were significantly lower in the con_HVD group than in the con_LVD group, indicating that vitamin D also modulates amyloidogenic gene expression under non-diabetic conditions.

**Figure 3:**
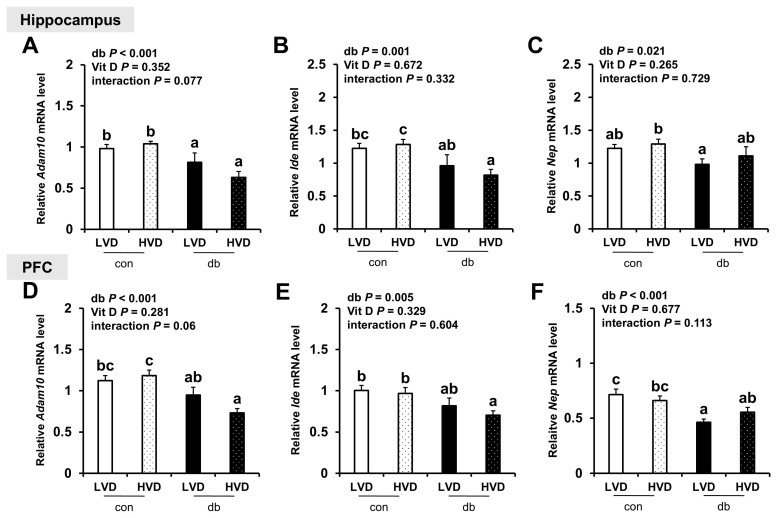
mRNA Expression Levels of Aβ Degradation-Related Genes in the Hippocampus and PFC. The relative mRNA expression levels of *Adam10*, *Ide*, and *Nep* in the hippocampus (**A**–**C**), along with those in the PFC (**D**–**F**), were measured using RT-PCR. Data are presented as mean values ± SEMs (n = 10 for con_LVD and con_HVD; n = 7 for db_LVD and db_HVD). *p*-values from two-way ANOVA are shown in the upper left corner of the graph to indicate the effects of diabetes, vitamin D levels, and their interaction. Different superscript letters (a, b, c) represent significant group differences (*p* < 0.05, as determined by Fisher’s LSD multiple-comparison test.

Regarding the expression of Aβ degradation-related genes (*Adam10*, *Ide*, and *Nep*), their levels were generally lower in the hippocampus and PFC of db/db mice (two-way ANOVA, *p* < 0.05); however, dietary vitamin D levels did not markedly affect their expression in either control or diabetic mice (Figure 3A–F).

**Figure 4:**
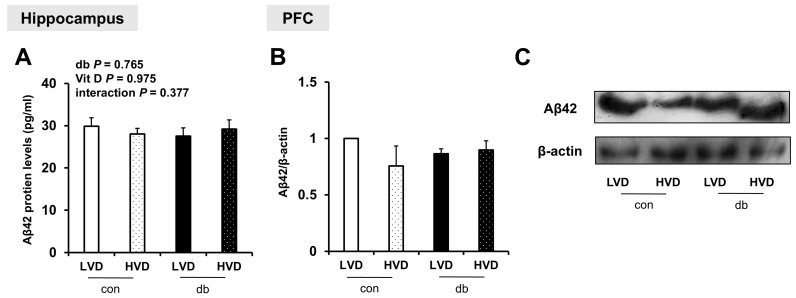
Aβ42 Protein Levels in the Hippocampus and PFC. (**A**) Aβ42 protein levels measured using ELISA in the hippocampus. Data are presented as mean values ± SEMs (n = 5–6 animals/group). *p*-values from two-way ANOVA are shown in the upper left corner of the graph to indicate the effects of diabetes, vitamin D levels, and their interaction. (**B**,**C**) Aβ42 protein levels in the PFC assessed using Western blotting. Densitometry results for Aβ42 expression normalized to β-actin. Data are presented as mean values ± SEMs (n = 2 animals/group).

To examine whether Aβ protein was accumulated in the hippocampus and PFC, and whether its levels were affected by diabetes and dietary vitamin D levels, Aβ42 protein levels were measured. Despite the upregulation of amyloidogenic genes in db/db mice and their suppression by high dietary vitamin D level, Aβ42 protein levels were not significantly affected by either diabetes or dietary vitamin D levels in the hippocampus or PFC (Figure 4A–C and Figure S2).

**Figure 5:**
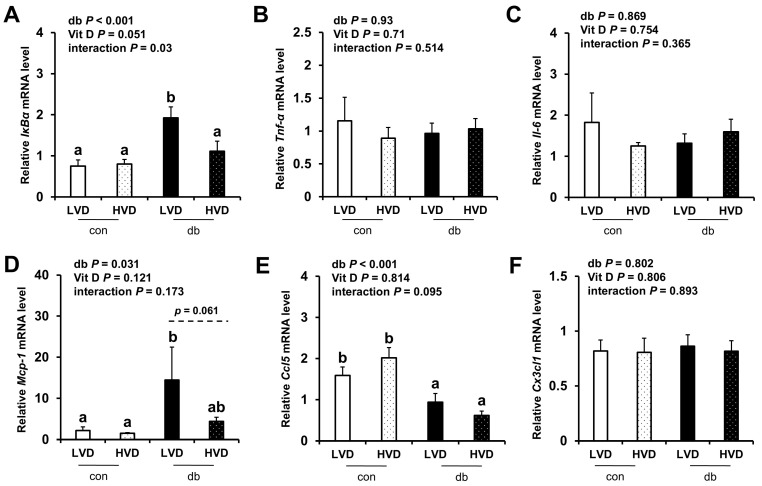
mRNA Expression Levels of Inflammatory Markers in the Hippocampus. The relative mRNA expression levels of *IκBα*, *Tnf-α*, *Il-6*, *Mcp-1*, *Ccl5*, and *Cx3cl1* in the hippocampus (**A**–**F**) were measured using RT-PCR. Data are presented as mean values ± SEMs (n = 10 for con_LVD and con_HVD; n = 7 for db_LVD and db_HVD). *p*-values from two-way ANOVA are shown in the upper left corner of the graph to indicate the effects of diabetes, vitamin D levels, and their interaction. Different superscript letters (a, b) represent significant group differences (*p* < 0.05), and *p*-value on the dashed line indicate difference between two groups, as determined by Fisher’s LSD multiple-comparison test.

**Figure 6:**
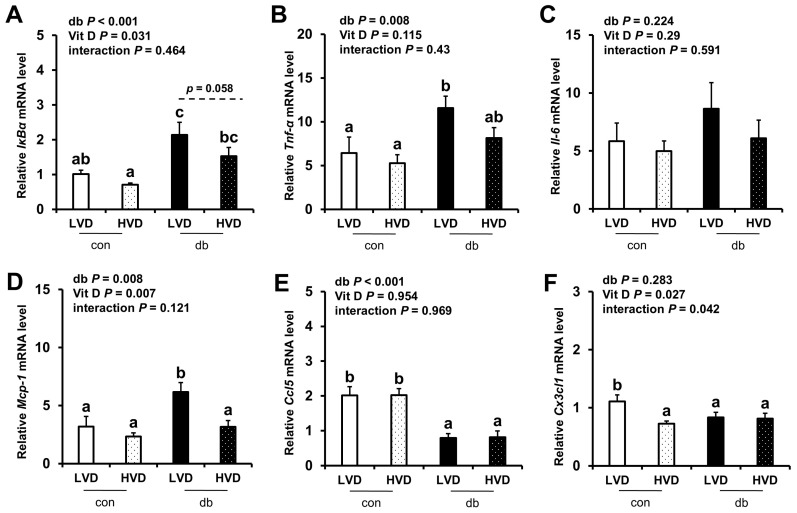
mRNA Expression Levels of Inflammatory Markers in the PFC. The relative mRNA expression levels of *IκBα*, *Tnf-α*, *Il-6*, *Mcp-1*, *Ccl5*, and *Cx3cl1* in the PFC (**A**–**F**) were measured using RT-PCR. Data are presented as mean values ± SEMs (n = 10 for con_LVD and con_HVD; n = 7 for db_LVD and db_HVD). *p*-values from two-way ANOVA are shown in the upper left corner of the graph to indicate the effects of diabetes, vitamin D levels, and their interaction. Different superscript letters (a, b, c) represent significant group differences (*p* < 0.05), and *p*-value on the dashed line indicate difference between two groups, as determined by Fisher’s LSD multiple-comparison test.

Given the observed reduction in neuronal cell death in the db_HVD group, we subsequently investigated whether different dietary vitamin D levels modulate diabetes-induced inflammation. We analyzed the mRNA expression levels of inflammatory cytokine-related genes (*IκBα*, *Tnf-α*, *Il-6*, *Mcp-1*, *Ccl5*, and *Cx3cl1*) in the hippocampus (Figure 5) and PFC (Figure 6). Overall, diabetic mice exhibited higher expression levels of hippocampal *IκBα* and *Mcp-1*, as well as PFC *IκBα*, *Tnf-α*, and *Mcp-1* (two-way ANOVA, *p* < 0.05). In db/db mice receiving a high levels of dietary vitamin D, a reduction in inflammation-related gene expression was observed; specifically, hippocampal *IκBα* and PFC *Mcp-1* expression levels were significantly lower (*p* < 0.05), while hippocampal *Mcp-1* (*p* = 0.061) and PFC *IκBα* (*p* = 0.058) tended to be lower compared to the db_LVD group. In contrast, *Ccl5* expression was lower across both brain regions in diabetic mice (*p* < 0.001), with no effect of dietary vitamin D levels. *Tnf-α* expression in the PFC was significantly higher in db_LVD mice than in their con_LVD counterparts (*p* < 0.05), while no significant changes were observed for *Tnf-α* in the hippocampus or *Il-6* and *Cx3cl1* in either region.

**Figure 7:**
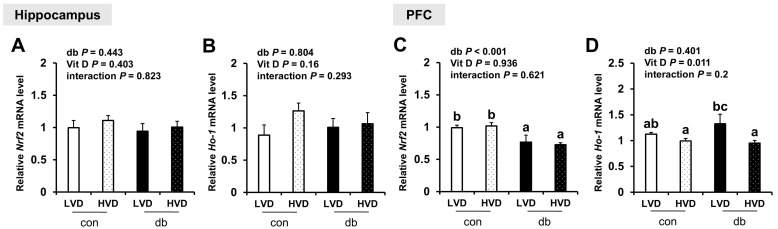
mRNA Expression Levels of Oxidative Stress-Related Genes in the Hippocampus and PFC. The relative mRNA expression levels of *Nrf2* and *Ho-1* in the hippocampus (**A**,**B**), along with those in the PFC (**C**,**D**), were measured using RT-PCR. Data are presented as mean values ± SEMs (n = 10 for con_LVD and con_HVD; n = 7 for db_LVD and db_HVD). *p*-values from two-way ANOVA are shown in the upper left corner of the graph to indicate the effects of diabetes, vitamin D levels, and their interaction. Different superscript letters (a, b, c) represent significant group differences (*p* < 0.05), as determined by Fisher’s LSD multiple-comparison test.

To further assess oxidative stress, we evaluated the mRNA expression levels of antioxidant response-related genes (*Nrf2* and *Ho-1*). In diabetic mice, *Nrf2* expression was significantly reduced in the PFC (two-way ANOVA, *p* < 0.05), whereas hippocampal *Nrf2* and *Ho-1* in both the hippocampus and PFC were not affected by diabetes. Overall, dietary vitamin D did not exert a significant effect on *Nrf* expression; however, among db/db mice, *Ho-1* expression in the PFC was significantly lower in db_HVD mice compared with in db_LVD mice (Figure 7, *p* < 0.05).

**Figure 8:**
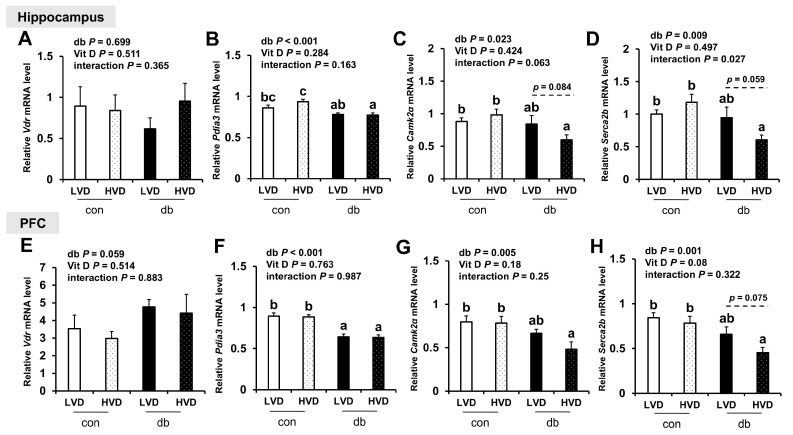
mRNA Expression Levels of Genes Related to Vitamin D Signaling and Calcium Homeostasis in the Hippocampus and PFC. The relative mRNA expression levels of *Vdr*, *Pdia3*, *CaMKIIα*, and *Serca2b* in the hippocampus (**A**–**D**), along with those in the PFC (**E**–**H**), were measured using RT-PCR. Data are presented as mean values ± SEMs (n = 10 for con_LVD and con_HVD; n = 7 for db_LVD and db_HVD). *p*-values from two-way ANOVA are shown in the upper left corner of the graph to indicate the effects of diabetes, vitamin D levels, and their interaction. Different superscript letters (a, b, c) represent significant group differences (*p* < 0.05), and *p*-values on the dashed line indicate difference between two groups, as determined by Fisher’s LSD multiple-comparison test.

To explore the mechanisms by which vitamin D operates through genomic (VDR) and non-genomic (PDIA3) pathways, as well as calcium homeostasis regulation, we measured the mRNA levels of *Vdr*, *Pdia3*, *CaMKIIα*, and *Serca2b* in the hippocampus and PFC (Figure 8). *Pdia3* expression was significantly downregulated in both brain regions of db/db mice compared with control mice (two-way ANOVA, *p* < 0.001), whereas *Vdr* expression remained unchanged. Dietary vitamin D levels did not significantly alter *Vdr* or *Pdia3* expression in either brain region. The expression levels of *CaMKIIα* and *Serca2b*, key regulators of calcium homeostasis, were significantly lower in db/db mice compared with those in control mice (two-way ANOVA, *p* < 0.05); however, no significant differences were observed between con_LVD and db_LVD mice. Hippocampal *CaMKIIα* and *Serca2b* expression, as well as that of *Serca2b* in the PFC, were lower in the db_HVD group compared with the db_LVD group, but the differences were not statistically significant.

**Figure 9:**
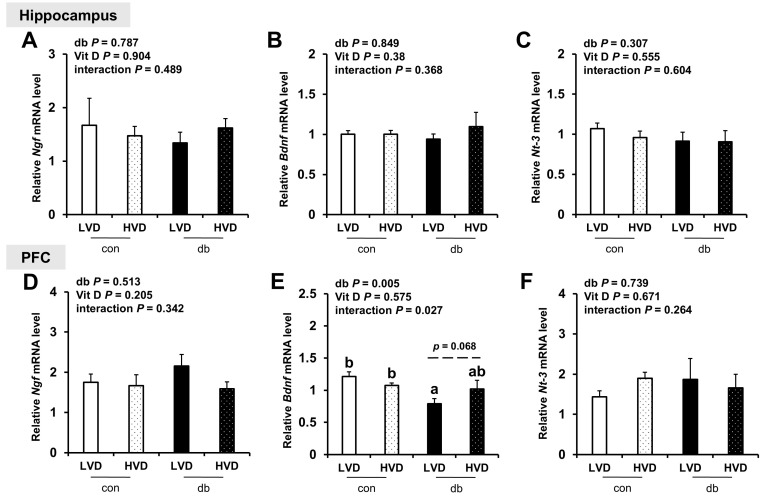
mRNA Expression Levels of Neurotrophic Factor-Related Genes in the Hippocampus and PFC. The relative mRNA expression levels of (**A**) *Ngf*, (**B**) *Bdnf,* and (**C**) *Nt-3* in the hippocampus, along with those of (**D**) *Ngf*, (**E**) *Bdnf*, and (**F**) *Nt-3* in the PFC, were measured using RT-PCR. Data are presented as mean values ± SEMs (n = 10 for con_LVD and con_HVD; n = 7 for db_LVD and db_HVD). *p*-values from two-way ANOVA are shown in the upper left corner of the graph to indicate the effects of diabetes, vitamin D levels, and their interaction. Different superscript letters (a, b) represent significant group differences (*p* < 0.05), and *p*-value on the dashed line indicate difference between two groups, as determined by Fisher’s LSD multiple-comparison test.

Considering the reduction in neuronal cell death in db_HVD group (Figure 1), we investigated its potential effects on neurotrophic factor expression (*Ngf*, *Bdnf*, and *Nt-3*; Figure 9. Among these, only PFC *Bdnf*, which was significantly lower in db/db mice (db_LVD), showed a tendency toward higher levels with a high dietary vitamin D level, but the change was not statistically significant (db_HVD, *p* = 0.068; Figure 9E). *Ngf* and *Nt-3* expression remained unchanged in both the hippocampus and PFC.

## 4. Discussion

This study examined the potential impact of dietary vitamin D levels on early AD-like pathology in db/db mice, a model of T2DM. Our key findings indicate that a higher dietary vitamin D level was associated with reduced neuronal apoptosis, relatively downregulated the expression of amyloidogenic genes (*App*, *Bace1*, and *Ps1*), and modulated inflammatory gene expression (*Mcp-1* and *IκBα*) in the hippocampus and PFC of db/db mice. These results suggest that vitamin D may confer protective effects against diabetes-induced neurodegeneration by targeting multiple pathological pathways.

The expression levels of amyloidogenic pathway-related genes (*App*, *Bace1*, and *Ps1*) in both the hippocampus and PFC were significantly lower in the db_HVD group than in the db_LVD group, suggesting that high doses of dietary vitamin D level partially attenuate the diabetes-induced upregulation of these genes and may help mitigate the activation of the amyloidogenic pathway. Similarly, in control mice, *App* and *Ps1* expression levels in the PFC are significantly lower in the con_HVD group compared with those in the con_LVD group, indicating that vitamin D may exert amyloidogenesis-modulating effects even under non-diabetic conditions. However, despite these transcriptional changes, Aβ42 protein levels in hippocampus and PFC did not change in response to diabetes or vitamin D.

This discrepancy suggests that Aβ accumulation is influenced not only by expression of amyloidogenic genes but also by other factors, such as the activity of degrading enzymes (Ide and Nep) [36], clearance mechanisms [37], and post-translational modifications [38]. In fact, the expression levels of Aβ degradation- or clearance-related genes (*Ide*, *Nep*, and *Adam10*) exhibited no significant changes in this study, suggesting that the effect of vitamin D may be more focused on regulating Aβ production rather than its clearance. Furthermore, the mice in this study were 5 weeks old at baseline, and they were examined at 13 weeks of age following an 8-week experimental period. Previous studies have revealed that endogenous Aβ is barely detectable in the brains of mice during early development, with substantial Aβ accumulation exclusively observed in aged mice, typically those aged over 60 weeks [39,40]. Consequently, transgenic mouse models that overexpress human Aβ are commonly employed to study Aβ deposition and plaque formation; however, these models may not accurately reflect the sporadic forms of AD experienced by most patients [39].

Taken together, the young age of the mice in the present study may account for the lack of significant differences in Aβ levels, despite alterations in amyloidogenesis-related gene expression. Additionally, a limitation of this study is the small sample size (n = 2/group) used for Western blot analyses of Aβ levels in the PFC, which may reduce the robustness of the protein-level validation. Future research involving older diabetic mice or transgenic models with higher Aβ accumulation is warranted to confirm these findings.

Notably, histological analyses utilizing H&E and Nissl staining revealed pronounced neuronal apoptosis in the CA1 region of db_LVD mice, which was markedly attenuated in the db_HVD group. These findings indicate that, even in the absence of detectable changes in Aβ42 protein levels, a higher dietary vitamin D level was associated with lower hippocampal neuronal loss in diabetic mice, suggesting potential neuroprotective effects. Collectively, these results imply that a higher dietary vitamin D level may preserve neuronal integrity through mechanisms that are at least partially independent of Aβ accumulation, underscoring its potential as a preventive strategy against diabetes-associated neurodegeneration.

Consistent with these neuroprotective effects, the higher *IκBα* expression observed in the db_LVD group compared with the db_HVD group suggests that vitamin D may modulate NF-κB signaling under diabetic conditions. *IκBα*, a critical inhibitor of NF-κB, typically suppresses pathway activation, yet its increased expression can also reflect a feedback response to NF-κB activation and ongoing inflammatory signaling [41,42]. In this context, the concurrent reduction in *Mcp-1* expression in the db_HVD group further corroborates the notion that vitamin D may attenuate neuroinflammation through complex mechanisms involving both NF-κB and downstream pathways such as MAPK [43,44]. Meanwhile, late-phase chemokines, such as Ccl5 and Cx3cl1, which are primarily induced by strong immune stimuli (e.g., IFN-γ) [45], were downregulated in db/db mice and remained unaffected by dietary vitamin D levels. Further investigation is needed to clarify the specific roles of each chemokine and to examine the effects of vitamin D in diabetes. Taken together, these findings suggest that vitamin D may mitigate diabetes-induced neuronal damage not only by reducing apoptosis but also by modulating key inflammatory pathways, supporting its potential role as a neuroprotective agent in diabetes-associated neurodegeneration.

The mRNA levels of antioxidant markers displayed brain region-specific differences in response to vitamin D. *Nrf2* expression in the PFC was significantly lower in db/db mice, regardless of vitamin D intake, indicating possible functional exhaustion under sustained oxidative stress [46,47]. The expression of the antioxidant marker *Ho-1* was higher in the db_LVD group than in the db_HVD group. Given that expression of *Ho-1* can also be regulated by the MAPK and NF-κB pathways [48,49], its lower expression in the db_HVD group may represent a compensatory response; however, further investigation is warranted.

Many prior studies have reported that vitamin D can increase the levels of neurotrophins, including nerve growth factor (NGF), glial cell-derived neurotrophic factor (GDNF), and brain-derived neurotrophic factor (BDNF), thus promoting neurogenesis and exerting neuroprotective effects [50,51]. In the current study, the mRNA expression levels of hippocampal *Ngf*, *Bdnf*, and *Nt-3* did not differ significantly among the experimental groups. *Bdnf* expression in the PFC significantly lower in the db_LVD group compared with that in the con_LVD group; however, the difference between the db_LVD and db_HVD groups was not statistically significant (*p* = 0.068). Several previous studies have reported that vitamin D can enhance BDNF expression by mitigating oxidative stress [52,53], suggesting a potential partial restoration of *Bdnf* expression following a high dietary vitamin D level.

In this study, mice received cholecalciferol through their diets; however, vitamin D regulates target genes via its biologically active form, 1,25-dihydroxyvitamin D, which mediates genomic actions through binding to nuclear VDRs and induces nongenomic responses through interactions with membrane-associated VDR or PDIA3 [54]. Neither dietary vitamin D level nor diabetes altered *Vdr* expression in the hippocampus or PFC. This finding is consistent with previous reports indicating that VDR primarily functions as a ligand-dependent transcription factor, with expression largely maintained across varying tissue vitamin D levels and predominantly regulated through ligand binding and post-translational modifications [55,56,57]. Conversely, *Pdia3* levels in the PFC were significantly lower in db/db mice compared with control mice, suggesting that chronic hyperglycemia and inflammation may affect non-genomic vitamin D signaling, potentially disrupting calcium regulation and protein homeostasis. Additional studies are warranted to clarify the specific roles of *Vdr* and *Pdia3* in mediating the effects of vitamin D on neuroinflammation and amyloidogenesis.

## 5. Conclusions

This study comprehensively assessed the impact of a high dietary vitamin D level (9477 IU/kg diet) on early AD-like pathology and the expression of related genes in db/db mice, a well-established T2DM model. A high dietary vitamin D level was associated with attenuated neuronal apoptosis in the CA1 region of the hippocampus and modulation of amyloidogenic genes (*App*, *Bace1*, and *Ps1*) and neuroinflammatory markers (*Mcp-1* and *IκBα*)—which had been upregulated under diabetic conditions—in both the hippocampus and PFC. In contrast, hippocampal and PFC Aβ protein levels were unaffected by diabetes or dietary vitamin D level, likely due to insufficient accumulation in the 13-week-old mice used. Overall, these findings support the potential of dietary vitamin D to mitigate early AD-like pathologies in a diabetic mouse model and underscore the imperativeness of further long-term, quantitative studies.

## Figures and Tables

**Table 1:** Body Weight, Food Intake, Vitamin D Intake, and Blood Glucose Levels in Experimental Mice ^1,2,3.^

	con		db		*p*-Value ^2^		
	con_LVD (n = 13)	con_HVD (n = 14)	db_LVD (n = 10)	db_HVD (n = 10)	Diabetes	Vit D Intake	Interaction
Body weight at 1 week (g)	17.9 ± 0.4 ^a^	18 ± 0.3 ^a^	27.1 ± 0.6 ^b^	27.7 ± 0.6 ^b^	<0.001	0.475	0.521
Body weight at 8 weeks (g)	25.1 ± 0.4 ^a^	25 ± 0.3 ^a^	34.1 ± 0.7 ^b^	33.1 ± 1.3 ^b^	<0.001	0.43	0.516
Average food intake (g/day)	2.6 ± 0.02 ^a^	2.5 ± 0.02 ^a^	5.1 ± 0.1 ^b^	5.1 ± 0.22 ^b^	<0.001	0.746	0.707
Average vitamin D intake (IU/day)	2.6 ± 0.02 ^a^	25.2 ± 0.23 ^b^	5.1 ± 0.1 ^a^	50.8 ± 2.19 ^c^	<0.001	<0.001	<0.001
Blood glucose level at 1 week (mg/dL)	186 ± 10.7 ^a^	195.2 ± 9.5 ^a^	541.3 ± 55.2 ^b^	577.4 ± 45.7 ^b^	<0.001	0.493	0.683
Blood glucose level at 8 weeks (mg/dL)	184.1 ± 9.8 ^a^	205.9 ± 12.2 ^a^	800.1 ± 22.1 ^b^	751.6 ± 31.7 ^b^	<0.001	0.485	0.07

^1^ Data are presented as mean values ± SEMs. ^2^ Two-way ANOVA was performed to assess the significant effects of diabetes and amounts of vitamin D, and their interaction. ^a,b,c^ Different superscript letters indicate significant differences (*p* < 0.05) among groups using Fisher’s LSD multiple-comparison test. ^3^ This table contains mice, some of which were previously reported in [35].

## Data Availability

The data presented in this study are available from the corresponding author on reasonable request.

## Supplementary Materials

The following supporting information can be downloaded at: https://www.mdpi.com/article/10.3390/nu17213339/s1, Figure S1. Anatomical Diagram of the Mouse Brain, Figure S2. Full-length and uncropped Western blot images of Figure 4C, Table S1. Composition of the Experimental Diets, Table S2. List of Primers Used for qRT-PCR in Mice.

## Abbreviations

ADAM10: A disintegrin and metalloproteinase domain 10; Aβ: Amyloid-beta; AD: Alzheimer’s disease; APP: Amyloid precursor protein; BACE1: Beta-site app cleaving enzyme 1; BDNF: Brain-derived neurotrophic factor; BBB: Blood–brain barrier; CAMKIIα: Calcium/calmodulin-dependent protein kinase II alpha; CCL5: Chemokine (C-C motif) ligand 5; CNS: Central nervous system; CSF: Cerebrospinal fluid; CX3CL1: Chemokine (C-X3-C motif) ligand 1; ELISA: Enzyme-Linked Immunosorbent Assay; GAPDH: Glyceraldehyde-3-phosphate dehydrogenase; GDNF: glial cell-derived neurotrophic factor; H&E: Hematoxylin and Eosin; HO-1: Heme oxygenase-1; HVD: high Vitamin D; IFN-γ: Interferon-gamma; IL-6: Interleukin-6; IDE: Insulin-degrading enzyme; IκBα: Inhibitor of nuclear factor kappa B alpha; JAK/STAT: Janus kinase / Signal transducer and activator of transcription; LVD: low Vitamin D; MAPK: Mitogen-activated protein kinase; MCP-1: Monocyte chemoattractant protein-1; NEP: Neprilysin; NF-κB: Nuclear factor kappa B; NGF: Nerve growth factor; NHANES: National Health and Nutrition Examination Survey; NRF2: Nuclear factor erythroid 2–related factor 2; NT-3: Neurotrophin-3; PDIA3: Protein disulfide isomerase family a member 3; PFC: Prefrontal cortex; PS1: Presenilin 1; qRT-PCR: Quantitative Reverse Transcription Polymerase Chain Reaction; SERCA2b: Sarco/endoplasmic reticulum calcium ATPase 2 beta; T2DM: Type 2 diabetes mellitus; TNF-α: Tumor necrosis factor alpha; VDR: Vitamin D receptor.

## References

[1] Gauthier S., Webster C., Servaes S., Morais J., Rosa-Neto P. (2023). World Alzheimer Report 2022: Life after Diagnosis: Navigating Treatment, Care and Support.

[2] Kinney J.W., Bemiller S.M., Murtishaw A.S., Leisgang A.M., Salazar A.M., Lamb B.T. (2018). Inflammation as a central mechanism in Alzheimer’s disease. Alzheimer’s Dement. Transl. Res. Clin. Interv..

[3] Kadowaki H., Nishitoh H., Urano F., Sadamitsu C., Matsuzawa A., Takeda K., Masutani H., Yodoi J., Urano Y., Nagano T. (2005). Amyloid beta induces neuronal cell death through ROS-mediated ASK1 activation. Cell Death Differ..

[4] Novoa C., Salazar P., Cisternas P., Gherardelli C., Vera-Salazar R., Zolezzi J.M., Inestrosa N.C. (2022). Inflammation context in Alzheimer’s disease, a relationship intricate to define. Biol. Res..

[5] Whitwell J.L. (2010). Progression of atrophy in Alzheimer’s disease and related disorders. Neurotox. Res..

[6] Chatterjee S., Mudher A. (2018). Alzheimer’s Disease and Type 2 Diabetes: A Critical Assessment of the Shared Pathological Traits. Front. Neurosci..

[7] Barbagallo M., Dominguez L.J. (2014). Type 2 diabetes mellitus and Alzheimer’s disease. World J. Diabetes.

[8] Xue M., Xu W., Ou Y.N., Cao X.P., Tan M.S., Tan L., Yu J.T. (2019). Diabetes mellitus and risks of cognitive impairment and dementia: A systematic review and meta-analysis of 144 prospective studies. Ageing Res. Rev..

[9] Rhea E.M., Banks W.A. (2021). A historical perspective on the interactions of insulin at the blood-brain barrier. J. Neuroendocrinol..

[10] Agrawal R., Reno C.M., Sharma S., Christensen C., Huang Y., Fisher S.J. (2021). Insulin action in the brain regulates both central and peripheral functions. Am. J. Physiol. Endocrinol. Metab..

[11] Banks W.A. (2004). The source of cerebral insulin. Eur. J. Pharmacol..

[12] Liu Y., Liu F., Grundke-Iqbal I., Iqbal K., Gong C.X. (2011). Deficient brain insulin signalling pathway in Alzheimer’s disease and diabetes. J. Pathol..

[13] Kim E.K., Choi E.J. (2010). Pathological roles of MAPK signaling pathways in human diseases. Biochim. Biophys. Acta.

[14] Gabbouj S., Ryhänen S., Marttinen M., Wittrahm R., Takalo M., Kemppainen S., Martiskainen H., Tanila H., Haapasalo A., Hiltunen M. (2019). Altered Insulin Signaling in Alzheimer’s Disease Brain—Special Emphasis on PI3K-Akt Pathway. Front. Neurosci..

[15] Bogush M., Heldt N.A., Persidsky Y. (2017). Blood Brain Barrier Injury in Diabetes: Unrecognized Effects on Brain and Cognition. J. Neuroimmune Pharmacol..

[16] Sutton A.L., MacDonald P.N. (2003). Vitamin D: More than a “bone-a-fide” hormone. Mol. Endocrinol..

[17] Ghaseminejad-Raeini A., Ghaderi A., Sharafi A., Nematollahi-Sani B., Moossavi M., Derakhshani A., Sarab G.A. (2023). Immunomodulatory actions of vitamin D in various immune-related disorders: A comprehensive review. Front. Immunol..

[18] L Bishop E., Ismailova A., Dimeloe S., Hewison M., White J.H. (2021). Vitamin D and Immune Regulation: Antibacterial, Antiviral, Anti-Inflammatory. JBMR Plus.

[19] Latimer C.S., Brewer L.D., Searcy J.L., Chen K.C., Popović J., Kraner S.D., Thibault O., Blalock E.M., Landfield P.W., Porter N.M. (2014). Vitamin D prevents cognitive decline and enhances hippocampal synaptic function in aging rats. Proc. Natl. Acad. Sci. USA.

[20] Mayne P.E., Burne T.H.J. (2019). Vitamin D in Synaptic Plasticity, Cognitive Function, and Neuropsychiatric Illness. Trends Neurosci..

[21] El-Atifi M., Dreyfus M., Berger F., Wion D. (2015). Expression of CYP2R1 and VDR in human brain pericytes: The neurovascular vitamin D autocrine/paracrine model. Neuroreport.

[22] Sailike B., Onzhanova Z., Akbay B., Tokay T., Molnár F. (2024). Vitamin D in Central Nervous System: Implications for Neurological Disorders. Int. J. Mol. Sci..

[23] Pinzon R.T., Handayani T., Wijaya V.O., Buana R.B. (2023). Low vitamin D serum levels as risk factor of Alzheimer’s disease: A systematic review and meta-analysis. Egypt. J. Neurol. Psychiatry Neurosurg..

[24] Annweiler C., Rolland Y., Schott A.M., Blain H., Vellas B., Herrmann F.R., Beauchet O. (2012). Higher vitamin D dietary intake is associated with lower risk of alzheimer’s disease: A 7-year follow-up. J. Gerontol. A Biol. Sci. Med. Sci..

[25] Chiu K.C., Chu A., Go V.L., Saad M.F. (2004). Hypovitaminosis D is associated with insulin resistance and beta cell dysfunction. Am. J. Clin. Nutr..

[26] Wamberg L., Christiansen T., Paulsen S.K., Fisker S., Rask P., Rejnmark L., Richelsen B., Pedersen S.B. (2013). Expression of vitamin D-metabolizing enzymes in human adipose tissue—The effect of obesity and diet-induced weight loss. Int. J. Obes..

[27] Scragg R., Sowers M., Bell C. (2004). Serum 25-hydroxyvitamin D, diabetes, and ethnicity in the Third National Health and Nutrition Examination Survey. Diabetes Care.

[28] Norris J.M., Lee H.S., Frederiksen B., Erlund I., Uusitalo U., Yang J., Lernmark Å., Simell O., Toppari J., Rewers M. (2018). Plasma 25-Hydroxyvitamin D Concentration and Risk of Islet Autoimmunity. Diabetes.

[29] Park C.Y., Shin S., Han S.N. (2024). Multifaceted Roles of Vitamin D for Diabetes: From Immunomodulatory Functions to Metabolic Regulations. Nutrients.

[30] Parker J., Hashmi O., Dutton D., Mavrodaris A., Stranges S., Kandala N.B., Clarke A., Franco O.H. (2010). Levels of vitamin D and cardiometabolic disorders: Systematic review and meta-analysis. Maturitas.

[31] Bharadwaj P., Wijesekara N., Liyanapathirana M., Newsholme P., Ittner L., Fraser P., Verdile G. (2017). The Link between Type 2 Diabetes and Neurodegeneration: Roles for Amyloid-β, Amylin, and Tau Proteins. J. Alzheimers Dis..

[32] Gu J.C., Wu Y.G., Huang W.G., Fan X.J., Chen X.H., Zhou B., Lin Z.J., Feng X.L. (2022). Effect of vitamin D on oxidative stress and serum inflammatory factors in the patients with type 2 diabetes. J. Clin. Lab. Anal..

[33] Manna P., Achari A.E., Jain S.K. (2017). Vitamin D supplementation inhibits oxidative stress and upregulate SIRT1/AMPK/GLUT4 cascade in high glucose-treated 3T3L1 adipocytes and in adipose tissue of high fat diet-fed diabetic mice. Arch. Biochem. Biophys..

[34] Mansournia M.A., Ostadmohammadi V., Doosti-Irani A., Ghayour-Mobarhan M., Ferns G., Akbari H., Ghaderi A., Talari H.R., Asemi Z. (2018). The Effects of Vitamin D Supplementation on Biomarkers of Inflammation and Oxidative Stress in Diabetic Patients: A Systematic Review and Meta-Analysis of Randomized Controlled Trials. Horm. Metab. Res..

[35] Oh M., Jung S., Kim Y.A., Lee G.Y., Han S.N. (2024). Dietary vitamin D(3) supplementation enhances splenic NK cell activity in healthy and diabetic male mice. Nutr. Res..

[36] Grimm M.O., Lehmann J., Mett J., Zimmer V.C., Grösgen S., Stahlmann C.P., Hundsdörfer B., Haupenthal V.J., Rothhaar T.L., Herr C. (2014). Impact of Vitamin D on amyloid precursor protein processing and amyloid-β peptide degradation in Alzheimer’s disease. Neurodegener. Dis..

[37] Yoon S.S., Jo S.A. (2012). Mechanisms of Amyloid-β Peptide Clearance: Potential Therapeutic Targets for Alzheimer’s Disease. Biomol. Ther..

[38] Grochowska K.M., Yuanxiang P., Bär J., Raman R., Brugal G., Sahu G., Schweizer M., Bikbaev A., Schilling S., Demuth H.U. (2017). Posttranslational modification impact on the mechanism by which amyloid-β induces synaptic dysfunction. EMBO Rep..

[39] Ahlemeyer B., Halupczok S., Rodenberg-Frank E., Valerius K.P., Baumgart-Vogt E. (2018). Endogenous Murine Amyloid-β Peptide Assembles into Aggregates in the Aged C57BL/6J Mouse Suggesting These Animals as a Model to Study Pathogenesis of Amyloid-β Plaque Formation. J. Alzheimers Dis..

[40] De Plano L.M., Saitta A., Oddo S., Caccamo A. (2024). Navigating Alzheimer’s Disease Mouse Models: Age-Related Pathology and Cognitive Deficits. Biomolecules.

[41] Guo Q., Jin Y., Chen X., Ye X., Shen X., Lin M., Zeng C., Zhou T., Zhang J. (2024). NF-κB in biology and targeted therapy: New insights and translational implications. Signal Transduct. Target. Ther..

[42] Downton P., Bagnall J.S., England H., Spiller D.G., Humphreys N.E., Jackson D.A., Paszek P., White M.R.H., Adamson A.D. (2023). Overexpression of IκB⍺ modulates NF-κB activation of inflammatory target gene expression. Front. Mol. Biosci..

[43] Ding C., Wilding J.P., Bing C. (2013). 1,25-dihydroxyvitamin D3 protects against macrophage-induced activation of NFκB and MAPK signalling and chemokine release in human adipocytes. PLoS ONE.

[44] Fenercioglu A.K., Gonen M.S., Uzun H., Sipahioglu N.T., Can G., Tas E., Kara Z., Ozkaya H.M., Atukeren P. (2023). The Association between Serum 25-Hydroxyvitamin D3 Levels and Pro-Inflammatory Markers in New-Onset Type 2 Diabetes Mellitus and Prediabetes. Biomolecules.

[45] Kawka E., Witowski J., Fouqet N., Tayama H., Bender T.O., Catar R., Dragun D., Jörres A. (2014). Regulation of chemokine CCL5 synthesis in human peritoneal fibroblasts: A key role of IFN-γ. Mediators Inflamm..

[46] Zhao J., Liu L., Li X., Zhang L., Lv J., Guo X., Chen H., Zhao T. (2019). Neuroprotective effects of an Nrf2 agonist on high glucose-induced damage in HT22 cells. Biol. Res..

[47] Sireesh D., Dhamodharan U., Ezhilarasi K., Vijay V., Ramkumar K.M. (2018). Association of NF-E2 Related Factor 2 (Nrf2) and inflammatory cytokines in recent onset Type 2 Diabetes Mellitus. Sci. Rep..

[48] Ryter S.W., Xi S., Hartsfield C.L., Choi A.M. (2002). Mitogen activated protein kinase (MAPK) pathway regulates heme oxygenase-1 gene expression by hypoxia in vascular cells. Antioxid. Redox Signal.

[49] Huang J., Guo P., Ma D., Lin X., Fang Q., Wang J. (2016). Overexpression of heme oxygenase-1 induced by constitutively activated NF-κB as a potential therapeutic target for activated B-cell-like diffuse large B-cell lymphoma. Int. J. Oncol..

[50] Fantini C., Corinaldesi C., Lenzi A., Migliaccio S., Crescioli C. (2023). Vitamin D as a Shield against Aging. Int. J. Mol. Sci..

[51] Wang W., Li Y., Meng X. (2023). Vitamin D and neurodegenerative diseases. Heliyon.

[52] Khairy E.Y., Attia M.M. (2021). Protective effects of vitamin D on neurophysiologic alterations in brain aging: Role of brain-derived neurotrophic factor (BDNF). Nutr. Neurosci..

[53] Mansouri F., Ghanbari H., Marefati N., Arab Z., Salmani H., Beheshti F., Hosseini M. (2021). Protective effects of vitamin D on learning and memory deficit induced by scopolamine in male rats: The roles of brain-derived neurotrophic factor and oxidative stress. Naunyn Schmiedebergs Arch. Pharmacol..

[54] Żmijewski M.A. (2022). Nongenomic Activities of Vitamin D. Nutrients.

[55] Oczkowicz M., Szymczyk B., Świątkiewicz M., Furgał-Dzierżuk I., Koseniuk A., Wierzbicka A., Steg A. (2021). Analysis of the effect of vitamin D supplementation and sex on Vdr, Cyp2r1 and Cyp27b1 gene expression in Wistar rats’ tissues. J. Steroid Biochem. Mol. Biol..

[56] Haussler M.R., Haussler C.A., Bartik L., Whitfield G.K., Hsieh J.C., Slater S., Jurutka P.W. (2008). Vitamin D receptor: Molecular signaling and actions of nutritional ligands in disease prevention. Nutr. Rev..

[57] Zenata O., Vrzal R. (2017). Fine tuning of vitamin D receptor (VDR) activity by post-transcriptional and post-translational modifications. Oncotarget.

