# Supplementary material for: Effects of Dietary Vitamin D Levels on Markers Related to Amyloidogenesis and Neuroinflammation in db/db Mice
Source: Nutrients. 2025 Oct 24;17(21):3339. doi: 10.3390/nu17213339 (PMC12608884; doi:10.3390/nu17213339)
Supplement: Supplementary file 1 [file nutrients-17-03339-s001.zip › nutrients-3918659-supplementary.pdf]

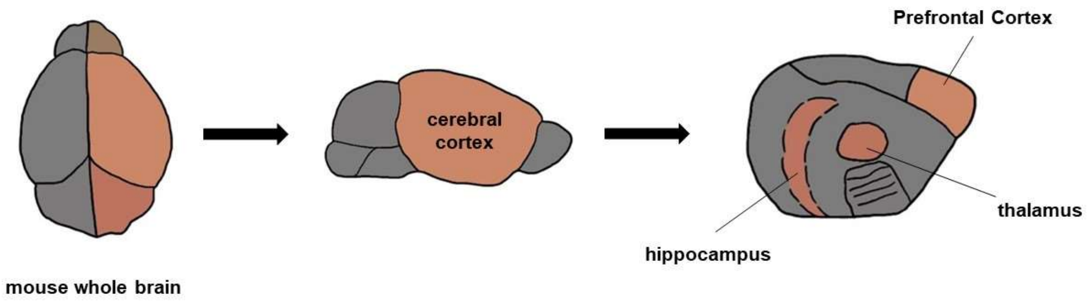

Figure S1. Anatomical Diagram of the Mouse Brain.

The diagram illustrates the major anatomical regions of the mouse brain. After extracting the forebrain, the brain was hemisected, and only the cerebral cortex was collected. The hippocampus, thalamus, and prefrontal cortex were then isolated from both the left and right hemispheres.

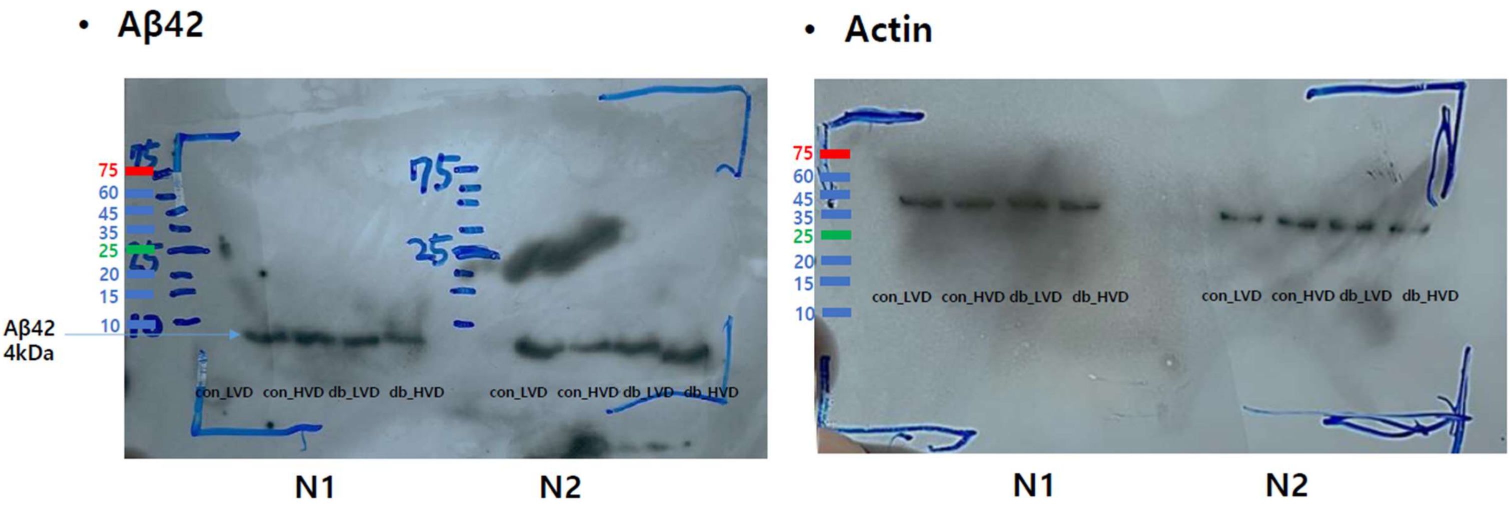

Figure S2. Full-length and uncropped Western blot images of Figures 4C

Western blot images of Aβ42 and β-actin proteins in PFC of con_LVD, con_HVD, db_LVD, and db_HVD groups.

Table S1. Composition of the experimental diets[1].

	LVD (948 IU vitamin D/kg diet)	HVD (9,477 IU vitamin D/kg diet)
Protein (kcal %)	20	20
Carbohydrate (kcal %)	70	70
Fat (kcal %)	10	10
Casein[2] (g)	200	200
L-Cystine (g)	3	3
Corn Starch (g)	452.2	452.2
Maltodextrin 10 (g)	75	75
Sucrose (g)	172.8	172.8
Cellulose, BW200 (g)	50	50
Soybean Oil (g)	25	25
Lard[3] (g)	20	20
Mineral Mix, S10026[4] (g)	10	10
Dicalcium Phosphate (g)	13	13
Calcium Carbonate (g)	5.5	5.5
Potassium Citrate, 1 H2O (g)	16.5	16.5
Vitamin Mix, V10001[5] (g)	10	10
Vitamin D3, 100,000 IU/g[6] (g)	0	0.09
Choline Bitartrate (g)	2	2
FD&C Yellow Dye #5 (g)	0.04	0
FD&C Red Dye #40 (g)	0.01	0
FD&C Blue Dye #1 (g)	0	0.05
Total (g)	1055.05	1055.14
kcal/g diet	3.8	3.8

[1]Resource: Research Diets Inc., New Brunswick, NJ, USA

[2]Casein is estimated to provide around 0.3 IU/g vitamin D

[3]Lard is estimated to provide around 0.2 IU/g vitamin D

[4]10 g of Mineral Mix (Research diets, Inc., #S10026) provides 1.0 g of Na, 1.6 g of Cl, 1.6 g of Mg, 0.33g of S, 59 mg of Fe, 29 mg of Zn, 6.0 mg of Cu, 2.0 mg of Cr, 1.6 mg of Mom 0.16 mg of Se, 0.9 mg of FI, 0.2 mg of I, and 3.99 g of sucrose

[5]10 g of Vitamin Mix (Research diets, Inc., #V10001) provides 4000 IU of vitamin A, 1000 IU of vitamin D3, 50 IU of vitamin E, 0.5 mg of menadione, 0.2 mg of biotin, 10 μg of vitamin B12, 2 mg of folic acid, 30 mg of niacin, 16 mg of pantothenic acid, 7 mg of vitamin B6, 6 mg of vitamin B2, 6 mg of vitamin B1, and 9.78 g of sucrose

[6]0.9 g of high-concentration Vitamin D3 (100,000 IU/g) was added only to the HVD diet to increase vitamin D content. This corresponds to an additional 9,000 IU (0.9 g × 100,000 IU/g).

Table S2. List of primers used for qRT-PCR in mice.

Gene Name	Orientation	Sequence
App	Forward	AGTGGTCAGTCCTCGGTCAG
	Reverse	TACGGAAACGACGCTCTCAT
Bace1	Forward	GATGGTGGACAACCTGAG
	Reverse	CTGGTAGTAGCGATGCAG
Ps1	Forward	AAGGTAATCCGTGGCGAAG
	Reverse	TGGAGACTGGAACACAACCA
Adam10	Forward	AGTGCCTGGAAGTGGTTTAGG
	Reverse	CATTGCTGAGTGGATTGTGG
Ide	Forward	CATAAAACCTCGGGCTCCTT
	Reverse	TCCCATACCAGACCTTCAGC
Nep	Forward	GCCTGGAACTCGGCTATGTA
	Reverse	CGGAAATCATCCCCCTAAAC
IκBα	Forward	CAGCATCTCCACTCCGTCCT
	Reverse	ACATCAGCCCCACATTTCA
Tnf-α	Forward	CTGGAAAGGTCTGAAGGTAGGAAGG
	Reverse	AACACAAGATGCTGGGACAGTGA
Il-6	Forward	CATTTCCACGATTTCCCAGAGA
	Reverse	TCCATCCAGTTGCCTTCTTGGG
Mcp-1	Forward	AGGCATCACAGTCCGAGTCAC
	Reverse	CCTTTTCCACAACCACCTCAAG
Ccl5	Forward	CTTGAACCCACTTCTTCTCTGG
	Reverse	TGCTGCTTTGCCTACCTCTC
Cx3cl1	Forward	GGAACCAACAAAGTCCGATG
	Reverse	TGAGAGTGAGGAAGCCAACC
Nrf2	Forward	GTCACTGGGCTCTGCTATGAA
	Reverse	TCTCCTCGCTGGAAAAAGAA
Ho-1	Forward	GGTGAGGGAACTGTGTCAGG
	Reverse	CAGGGGCTGTGAACTCTGTC
Vdr	Forward	GGGATGATGGGTAGGTTGTG
	Reverse	GGAAGAGGGTAGAGGGCAGA
Pdia3	Forward	GTGGCATCCATCTTGGCTAT
	Reverse	TCTGAACCCATCCCAGAGTC
Camk2α	Forward	TTTGAGGAACTGGGAAAGGG
	Reverse	CATGGAGTCGGACGATATTGG
Serca2b	Forward	AACCAAGCCAAAACGAAAGA
	Reverse	ACACAAAGACCGTGGAGGAG

Ngf	Forward	TGAGTGCCAAAAACAATCCA
	Reverse	GCGACCAGAGTCCCCTTTAT
Bdnf	Forward	CCTTACTATGGTTATTTCATACTTCGGTT
	Reverse	TCAGCCAGTGATGTCGTCGTC
Nt-3	Forward	ATGCCACGGAGATAAGCAAG
	Reverse	GCGAGACTGAATGACCGAAC
Gapdh	Forward	GGAGAAACCTGCCAAGTA
	Reverse	AAGAGTGGGAGTTGCTGTTG

APP: Amyloid precursor protein; BACE1: Beta-site app cleaving enzyme 1; BDNF: Brain-derived neurotrophic factor; CCL5: Chemokine (C-C motif) ligand 5; CX3CL1: Chemokine (C-X3-C motif) ligand 1; GAPDH: Glyceraldehyde-3-phosphate dehydrogenase; HO-1: Heme oxygenase-1; IL-6: Interleukin-6; IDE: Insulin-degrading enzyme; IκBα: Inhibitor of nuclear factor kappa B alpha; MCP-1: Monocyte chemoattractant protein-1; NEP: Neprilysin; NGF: Nerve growth factor; NRF2: Nuclear factor erythroid 2–related factor 2; NT-3: Neurotrophin-3; PDIA3: Protein disulfide isomerase family a member 3; PS1: Presenilin 1; SERCA2b: Sarco/endoplasmic reticulum calcium ATPase 2 beta; TNF-α: Tumor necrosis factor alpha; VDR: Vitamin D receptor.
